# Advances in numerical modelling of tyre fatigue performance: a review

**DOI:** 10.1186/s40712-025-00315-7

**Published:** 2025-08-19

**Authors:** Ayse Mujdeci, Veronica Marchante Rodriguez, Hasher Maqbool, Adam Amadeo, Marzio Grasso

**Affiliations:** 1 Faculty of Engineering and Applied Science, Cranfield University, Bedford, UK; 2 Dunlop Aircraft Tyres Ltd., 40 Fort Parkway, Birmingham, UK

**Keywords:** Tyres, Fatigue, Crack nucleation, Crack propagation, Finite element modelling

## Abstract

The growing emphasis on sustainability and environmental impact has driven increased demand for eco-friendly tyres. Tyre components are usually subjected to substantial static and dynamic load and often fail due to crack initiation and crack propagation. Understanding of the deformation mechanism of tyre components under fatigue loading is essential for enhancing the safety and reliability of tyres. In recent years, advanced tools for predicting fatigue and wear have been introduced, improving the accuracy of virtual prototyping and enabling more extensive evaluation of design concepts at early stages. This paper reviews recent advancements in the use of numerical methods for predicting fatigue failure and damage in tyre design. Given the limited research on numerical modelling for fatigue and fracture, there is a need for further investigation to develop reliable simulations for predicting tyre behaviour under fatigue loads. This review summarises the current applications of numerical fatigue modelling, providing engineers with a systematic overview of the literature, highlighting key achievements, and promoting further development in the field. The paper begins by discussing tyre components, followed by an exploration of material modelling techniques. It then addresses numerical modelling strategies for full-scale tyres under real-life loading conditions. Challenges in predicting fatigue failure using finite element (FE) modelling are examined, along with the issue of potential damage accumulation. Finally, the paper outlines recommendations for future research on FE modelling techniques, offering insights into current approaches and encouraging further investigation in the field.

## Introduction

Tyres, widely used across various sectors, are essential in the automotive and aircraft industries for their ability to endure extreme loading conditions, including high cyclic frequencies and large deformations, which contributes to improved comfort and vehicle performance (Shiraishi et al. 2006; Kongo Kondé et al. 2013; Chen 2022). The rapid growth and variety of vehicles worldwide are increasing the tyre production that is driving the development of sustainable and cost-effective solutions in response to environmental goals. Tyre lifetime is a critical consideration for designers due to its potential impact on costs. This includes increased man-hours, logistical challenges in acquiring and transporting replacement tyres, additional purchases, disposal expenses, and the intense competition within the tyre market. Currently, tyre specifications are outlined based on the required loads, speeds, inflation pressures, and flotation capabilities specific to the application platform in the tyre specifications including Technical Standard Order (TSO)-C62 (Service 2009), Society of Automotive Engineers (SAE) Aerospace Standard (AS)–4833 (Society of Automotive Engineers (SAE) Aerospace Standard (AS) 2014), and Military Performance Specification (Mil-PRF-5041) (Department of Defense (DoD) Department 2007). However, the actual performance of tyres under fatigue loading remains uncertain without experimental or numerical evaluations.

Fatigue refers to the initiation and propagation of cracks under variable stress conditions, which is crucial for tyres due to the deterioration in performance and accumulation of damage over time. This is especially relevant in scenarios where cyclic loading is common, such as taxing, take-offs, and landings in the case of aircraft tyres. In these conditions, tyres experience fluctuating loads that can lead to fatigue failure, highlighting the importance of ensuring their durability under demanding operating conditions.

**Fig. 1:**
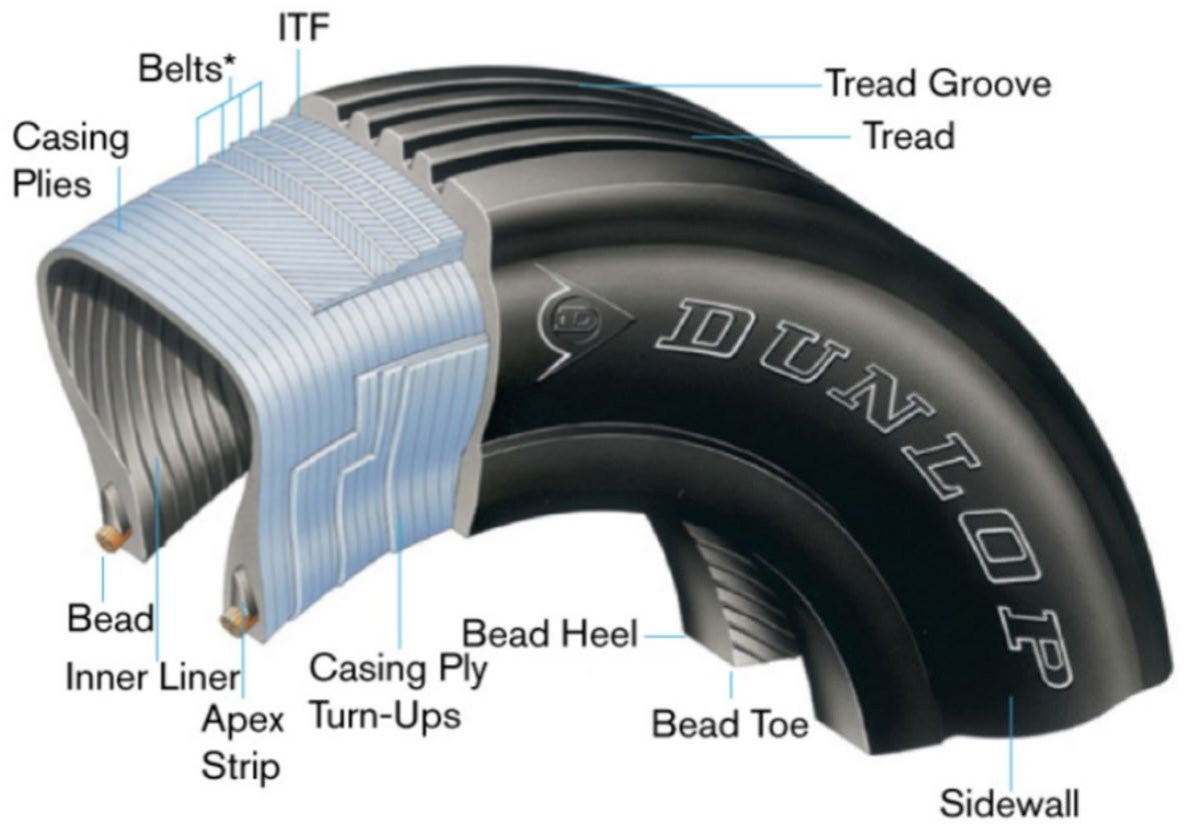
Tyre construction of radial tyres (Dunlop Aircraft Tyres Ltd. (n.d.))

Figure 1 illustrates the structure and main components of a typical radial tyre, namely body, sidewalls, beads, and treads. The tyre body is made from layers of rubberised fabric, known as plies, which provide both strength and flexibility. These plies are constructed from materials such as steel, rayon, nylon, or polyester cords, while chemically treated rubber is used for the sidewalls and tread. Additionally, beads are embedded in the inner edges of the tyre to support the rim. The fatigue performance of tyres is significantly influenced by the design and interaction of their components, necessitating a thorough evaluation of factors such as build geometry, thermal history, and interfacial relationships. Moreover, gaining a better understanding of the evolution of both thermal and mechanical failure at various stages of the service is important for effective design, especially when considering real-world conditions. Fatigue failure may occur due to defects like cuts, chips, or cracks in the rubber at the tyre’s belt edge (Giuliani et al. 2001; Xie et al. 2011; Gudsoorkar and Bindu 2020). The primary cause of such failure is the high shear stress resulting from the differing elastic properties of the layers, along with hydrostatic stress, particularly at the belt edge (Feng et al. 2004; Mars et al. 2018; Gudsoorkar and Bindu 2020). Reports also indicate that belt separation, carcass ply separation, and lug cracking are critical types of damage resulting from high amplitude loading and stiffness discontinuities (Ebbott 1996; Mars 2001; Han et al. 2004; Jeong et al. 2019). A detailed understanding of the fatigue and fracture behaviour of individual tyre components helps to advance durability and assess overall tyre performance.

Over the past few decades, numerous experimental studies have been conducted to investigate automotive and aircraft tyres. As the use of tyres has expanded across different applications, research activities have also grown, and the experimental data collected has helped to develop predictive trends regarding tyre lifespan (Xie et al. 2011; Shi et al. 2015; Li et al. 2017; Gudsoorkar and Bindu 2020). However, the experimental results and corresponding assessment were based on tested parameters specific to the tyre used. Therefore, the assessment is only valid under these conditions and may not be applicable if the parameters or sample type are changed. To address this limitation, numerical modelling provides a practical means to investigate a wider range of configurations and loading scenarios without the need for extensive physical testing. Moreover, effective design procedures require a comprehensive database that includes representative configurations of various material and geometric parameters, along with a wide range of loading conditions. Nonlinear computer modelling techniques can predict real-world behaviour under various loading scenarios and provide crucial information for developing reliable assessment and design procedures. As a result, these modelling techniques are often employed to complement experimental datasets. In terms of numerical tyre design and damage prediction using finite element (FE) simulations, accurate FE modelling is important for linking the tyre’s key components such as stress–strain relationships, viscoelasticity, fatigue crack growth, and interface behaviour. Therefore, detailed sensitivity studies using various numerical modelling strategies, using commercial software like Abaqus, Ansys, Fe-Safe, and Marc, have been conducted to evaluate new designs and predict the performance of tyre components or full tyres under fatigue loading (Kim et al. 2004; Zine et al. 2006, 2011; Boulenouar et al. 2016; Liu et al. 2023; Xing et al. 2024). Various numerical tools have been used to simulate tyre behaviour, and the complexity arising from the numerous nonlinear phenomena, including the tyre’s highly nonlinear behaviour under operating conditions, loading dynamics, frictional interactions, and numerous interrelated factors, has been documented. Most of the FE software offer an extensive library of material models, including hyperelastic, viscoelastic, creep, and damage models, along with nonlinear analysis that supports user-defined simulations through subroutines (e.g., UMAT, UEL). These features make commercial software like Abaqus the optimal choice to simulate complex nonlinear and hyperelastic behaviour of tyres (Behroozi 2014; Louis and Baumard 2016). However, previous numerical studies on the fatigue performance and fracture mechanisms of tyres have been limited by the complexity of the modelling techniques, as in outlined in Rubber fatigue characterisation section.

The aim of this review article is to critically assess existing research on fatigue criteria and tyre failure at both the component and tyre level. A comprehensive literature review has been conducted, covering over 82 publications, including journal articles and conference papers on fatigue failure mechanisms, as well as numerical modelling techniques used to simulate tyre components and applications under fatigue loading. It is worth noting that most of the work related to the development of numerical tools for predicting tyre fatigue and fracture is conducted by manufacturers and is not publicly available, as it represents specialised knowledge that provides a competitive advantage in the market. The ultimate goal of this review paper is to identify the most effective modelling approaches with an overview of current progress and a foundation for future works in tyre fatigue and fracture analysis.

## Rubber fatigue characterisation

**Fig. 2:**
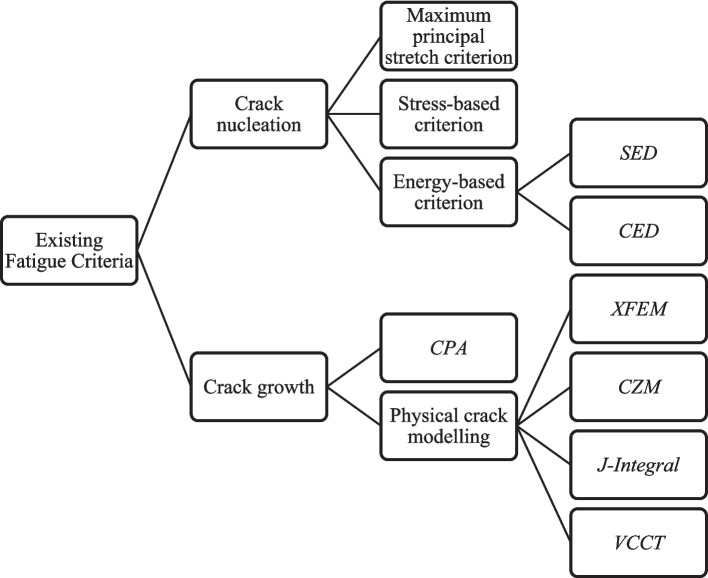
Existing fatigue criteria in the literature

Tyre components experience multiaxial fatigue loading, and their cyclic response is severe due to the complexity of the load paths. Investigating the cyclic stress–strain response under multiaxial loading is necessary for understanding crack mechanisms and ensuring structural integrity in design. The crack mechanism can be divided into three stages: (i) crack nucleation, (ii) crack propagation, and (iii) final failure. The first stage focuses on identifying the minimum requirements for crack initiation under cyclic loading. The second stage is the primary contributor to fatigue failure, as studying crack propagation helps estimate the remaining fatigue life. The final stage is key to ensuring material reliability and productivity in tyre design during service. A brief discussion of common fatigue criteria based on crack nucleation and crack growth approaches (Fig. 2), along with their predictive capabilities and application to rubber materials and tyres, is summarised in this review. This section characterises rubber fatigue behaviour from the perspective of crack nucleation and crack propagation. It outlines the key criteria and parameters—such as principal stretch, stress amplitude, and tearing energy that are commonly used in fatigue simulations of tyre and rubber components. These parameters form the basis for implementing damage models in numerical simulations, as discussed in Finite element models section.

### Crack nucleation

In recent years, several approaches have been implemented to study tyre durability. These include (i) continuum-based approaches, which assess stress–strain limits to define crack initiation life (Ayoub et al. 2011, 2012); and (ii) approaches that introduce an explicit crack feature to investigate crack propagation life, based on the strain energy release rate in fracture mechanics theory (Ebbott 1996; Wei et al. 1999; Han et al. 2004; Ozelo et al. 2012).

#### Maximum principal stretch criterion

Fatigue crack initiation is consequence of exceeding the critical deformation thresholds under cyclic loading. The maximum principal stretch criterion (*λ*) is a kinematic measure, and it has been used as a practical indicator in assessing fatigue behaviour in rubber materials. *λ* can be used as an indication of early-stage damage prediction; however, *λ* does not directly link to the fracture toughness (such as tearing energy or *J-integral*). The maximum principal stretch criterion, formulated by Kawabata (1973), is a modification of the Rankine criterion, where the principal stress is replaced by the maximum principal stretch. The maximum principal stretch is defined in Eq. (1):

1 $$\lambda=\frac L{L_0}$$

where *λ* is the stretch ratio, *L*_*0*_ is initial length of material, and* L* is final length of material. The maximum principal stretch criterion has been widely applied in tyre design, though often without consideration for performance under multiaxial loading conditions, such as combined tension and torsion (Ayoub et al. 2014). Cadwell et al. (1940) studied the fatigue life of natural rubber (NR) and established a correlation between the minimum strain at specific strain amplitudes and fatigue life. Fielding (1943) similarly found that increasing minimum strain improves the fatigue life of natural rubber when subjected to constant strain amplitude. Mars (2001) emphasised that multiaxial behaviour and damage accumulation over time are key factors in fatigue analysis, and relying on the maximum principal stretch criterion may oversimplify the complex behaviour seen in multiaxial fatigue loading. Additionally, this approach has limitations under compressive loading because it neglects crack closure. Ayoub et al. (2014) demonstrated that the maximum principal stretch approach fails to accurately predict fatigue life in styrene-butadiene rubber (SBR) samples across different loading conditions, including tension, torsion, and combined tension–torsion. Their findings indicated that while predictions for tension and combined tension–torsion tests were consistent, torsion test predictions tended to overestimate fatigue life.

Green–Lagrange strain approach was utilised by Kim et al. (2004) and Woo et al. (2009) to predict the fatigue life of NR diabolo samples. Their studies highlighted that the maximum principal strain approach provides better correlation with experimental results than the strain energy density approach. Despite its widespread use, the maximum principal strain approach may not always be suitable for comprehensive fatigue analysis, as it may fail to fully capture the complexities required to unify multiaxial data.

#### Stress-based criterion

Another common approach for analysing materials and structures under fatigue loading is the stress-based criterion, which uses applied stresses to predict resistance until failure (Wang et al. 2002; Ayoub et al. 2011, 2014; Tee et al. 2018). Failure occurs when the applied stress exceeds critical levels. Wang et al. (2002) and Miehe (1995) demonstrated that the lifespan of elastomers can be predicted using continuum damage mechanics (CDM). Saintier et al. (2006) applied the Cauchy stress tensor method to evaluate the multiaxial stress states of rubbery materials. Their results, presented in a Haigh-Soderberg diagram, used the mean and amplitude of Cauchy stress under push–pull loading conditions. Using the Cauchy stress tensor to predict the fatigue life of natural rubber (NR) was not effective. Additionally, it is reported that the stress-based criterion is more general and applicable to a wide range of materials; however, it results in relatively inaccurate outcomes that are inconvenient for elastomers. Abraham et al. (2005) reported that the stress-based criterion is inadequate for predicting the fatigue life of elastomers, especially for non-strain-crystallising rubber. Similarly, Alshuth et al. (2002) reported that the lifetime of rubber decreases with zero minimum stress and varying stress amplitude. Moreover, the stress-based approach is limited in its ability to identify damage, as it relies on the definition of a damage evolution law, which may not fully address the estimation of fatigue life, especially when crack propagation occurs. To provide a more reliable assessment of tyre fatigue life, a detailed understanding of crack propagation, including factors such as crack growth, size, and shape, is essential. As a result, using stress-based criterion in fatigue analysis can offer limited advantages.

#### Energy-based criterion

The energy-based approach is commonly used to assess fatigue failure through energy absorption or dissipation under loading. Strain energy density (*SED*) offers a simplified method, where the energy release rate is directly proportional to both the *SED* and crack size. This approach has been adapted to define FE material models in Abaqus, taking into account various parameters such as material testing methods, continuum mechanics principles, and microstructural responses. However, it has been reported that the strain energy approach cannot unify uniaxial and equiaxial tensile behaviour (Ayoub et al. 2014), and it is also unable to predict uniaxial tensile and pure shear performance accurately (Tee et al. 2018). Additionally, this approach does not account for the orientation of nucleated cracks.

**Fig. 3:**
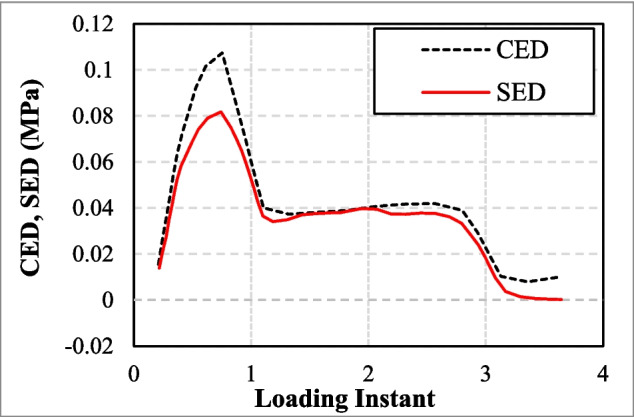
A comparison between strain and cracking energy densities for a particular failure plane in (Nyaaba et al. 2019a)

In an attempt to improve the *SED* method, a theoretical framework based on crack energy density (*CED*) was proposed by Mars (2002) to capture the unified multiaxial fatigue behaviour to predict the stored energy in a specified material plane under complex strain histories. This method provides the energy available for crack initiation on each potential plane, focusing on the local strain energy and the contribution of each plane to the overall damage. It is essential to investigate the energy density of a given strain state as well as the crack orientation regarding fatigue crack initiation life (Mars 2002). A comprehensive investigation into the applicability of *CED* was conducted and further expanded in Harbour et al. (2008), Ayoub et al. (2011), and Zine et al. (2011), with the method also being integrated into Configurational Mechanics Theory (Ayoub et al. 2011, 2012). Nyaaba et al. (2019a) employed *CED* parameter, a subset of the total strain energy density, to capture the damaging impact of multiaxial loads on all potential cracking planes. They reported that *CED* function adjusts for multiaxial loading conditions while considering the strain energy distribution across different planes under complex loading. A comparison was made between strain energy density with cracking energy density for a specific failure plane, showing that the strain energy available for release on the cracking plane is limited under multiaxial strain conditions (Fig. 3).

### Crack growth

While many procedures and approaches have been proposed to investigate crack nucleation, it is equally important to consider crack growth. Crack propagation accounts for a significant portion of a component’s fatigue life and offers a more comprehensive approach, as it considers cumulative damage under applied deformations or loading, unlike methods focused only on crack nucleation.

#### Critical plane analysis

Critical plane analysis (*CPA*) establishes a correlation between the number of cycles and crack orientation to identify failure on each failure plane. This ‘critical plane’ is where the combined effects of stress, strain, or energy are most likely to initiate and propagate a crack. *CPA* involves assessing the number of cycles needed for a crack precursor to form on each potential failure plane and then determining the plane with the shortest expected lifespan (Mars 2002; Barbash and Mars 2016).

**Fig. 4:**
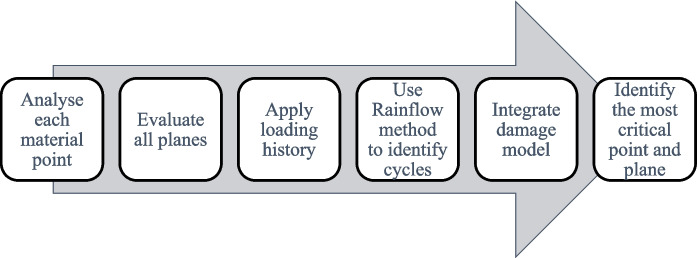
Fatigue life prediction for CPA algorithm

The *CPA* algorithm (Fig. 4) and energy release rate analysis have been used to predict fatigue failure and crack growth in elastomers by the authors Mars and Fatemi 2003), Barbash and Mars (2016), and Mars (2021). The most widely applied numerical method for fatigue crack growth in rubber is the critical plane approach, which utilises incremental fatigue analysis and is available in commercial software packages such as Endurica CL (Mars 2021). This approach enables the identification of damage levels across various temperature conditions. Key parameters such as fatigue lifetime, crack growth rate (*da/dN*, where ‘*a*’ is the crack tip position and ‘*N*’ is the number of cycles), and the loading ratio are central to the analysis. Damage evolution, indicated by crack length increase, is assessed using energy release rate, while tear energy fatigue limits were incorporated to identify transition points in the rate law. Unlike other methods, *CPA* does not require the explicit meshing of cracks in FE models after constructing the tyre model using standard procedures. This makes it advantageous in avoiding the computational costs and convergence issues often associated with meshed cracks in FE models (Mars et al. 2019). Additionally, *CPA* is broad in its application and offers the significant benefit of not needing a pre-inserted crack in the meshed geometry. This approach provides extensive insights into the durability and failure mechanics of the bushing with relatively minimal analysis effort (Mars 2021). However, there is a need for more detailed modelling approaches that can account for factors such as ageing, cyclic softening, inelasticity, rate effects, and anisotropy to fully understand the ultimate fatigue failure conditions.

#### Physical crack modelling approaches

Physical crack modelling approaches are commonly used to simulate crack initiation, growth, and the interaction of cracks within the material. A fracture criterion based on the energy release rate concept is frequently applied in crack growth modelling. However, to accurately predict the direction of crack propagation, some modelling techniques require the identification of initial crack locations and path.

Extended finite element method (*X-FEM*) is able to model the crack growth without requiring the definition of the crack path a priori, unlike other methods that require mesh adjustments to align with the crack edges, especially in regions with discontinuous stress and displacement fields (Sukumar et al. 2003; Gigliotti and Kroon 2012; Behroozinia et al. 2019). By integrating enrichment functions, the *X-FEM* approach allows cracks to pass through elements arbitrarily, enabling the mesh to remain consistent throughout crack development despite variations in field characteristics (Stolarska et al. 2001). *X-FEM* has been employed in various studies to predict the fatigue life of rubbers and ply-rubber composite materials (Behroozinia et al. 2016, 2019).

**Fig. 5:**
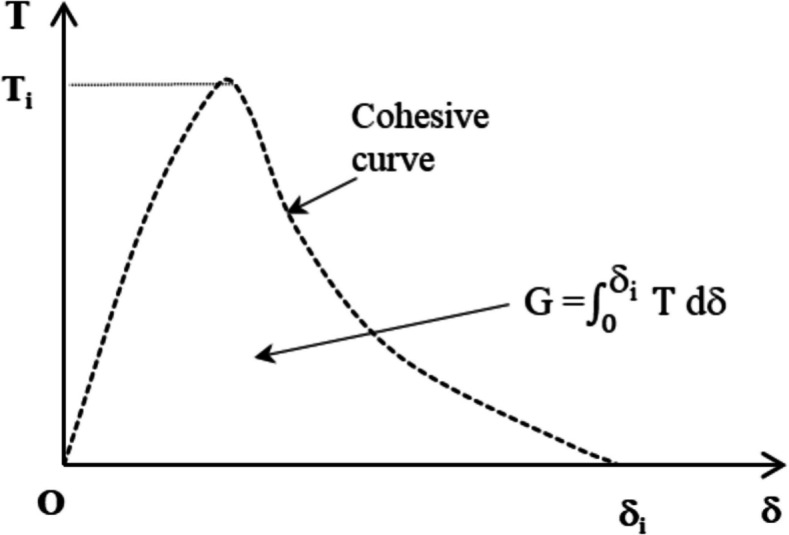
Traction–separation law of CZM (Li and Hoo Fatt 2015)

The cohesive zone modelling (*CZM*) approach is used to simulate crack initiation and propagation in materials by introducing a traction–separation law in cohesive zone elements. *CZM* can also assess the fracture between two adhesive surfaces under dynamic loading (Li and Hoo Fatt 2015). The traction–separation curve is defined by three parameters: peak traction (*T*_*i*_), maximum displacement (*δ*_*f*_), and energy release rate (*G*) (Fig. 5). It is important to note that in practical applications, *G* is indirectly considered through the definitions of *T*_*i*_ and δ_f_. *CZM* operates in three phases to investigate hyper-viscoelastic behaviour: (i) before damage, (ii) damage initiation based on critical strain energy density, and (iii) after damage initiation. However, *CZM* is sensitive to mesh density, as fine mesh modelling is often required in specific regions, which negatively affects computational efficiency while still accurately capturing the crack mechanism (Li and Hoo Fatt 2015).

The *J-integral* approach is calculated as the amount of energy released per unit area of crack surface growth under large deformations. In this context, the strain energy release rate is used to define *J-integral* equations, incorporating stress outputs from planar material tests, such as tensile, simple shear, or combined tensile and shear tests by Bhattacharyya et al. (2023). However, this method focuses on the crack initiation and neglects the new crack surfaces in tyre; meanwhile, it leads to the stress singularity problem (Li and Hoo Fatt 2015).

**Fig. 6:**
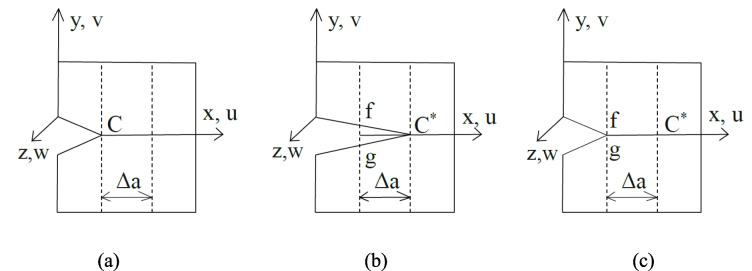
(**a**) Schematic crack propagation diagram of VCCT showing the crack initiation, (**b**) crack propagation, and (**c**) closure of the crack (Kim et al. 2005)

Virtual crack closure technique (*VCCT*) method has been implemented using fracture energy density or strain energy release rate to assess crack propagation direction by considering the total energy released (Kim et al. 2005). After a crack is introduced on the plane (Fig. 6(a)), it extends by a length Δa, separating two nodes (f and g) as shown in Fig. 6(b). To return these two nodes to their original positions, the same amount of energy is released as the crack propagates Fig. 6(c). This technique requires complex algorithms to track the crack tip and allow propagation by removing constraints on duplicate nodes. As a result, the propagation path must be identified by using 2D numerical models with one or two crack fronts (Harper and Hallett 2010). However, only a limited number of studies have focused on *VCCT* for rubber and cord-rubber materials. It has been noted that failure locations and crack propagation directions can vary, especially in critical areas such as the bead and shoulder regions of the tyre. As a result, the *VCCT* method may not be suitable for examining failure patterns in tyres under fatigue loading (Liang et al. 2021).

Based on the review of existing studies, the key conclusions are summarised in Table 1:

**Table 1:** Summary of previous studies into various damage levels

Approach	Method	Advantages	Disadvantage	Key References
Crack nucleation	Maximum principal stretch criterion	- Widely used in tyre design applications - Simple approach	- Simplifies complex behaviour under multiaxial loading - Not reliable under compressive loading due to the neglect of crack closure - Provides limited fracture data on early-stage damage prediction	- (Cadwell et al. 1940; Fielding 1943; Mars 2001; Kim et al. 2004; Woo et al. 2009; Ayoub et al. 2014)
	Stress-based method	- Captures unified multiaxial fatigue behaviour - Applicable to a wide range of materials	- Results can be relatively inaccurate for elastomers when minimum stress is zero - Limited in addressing fatigue life, especially crack propagation	- (Miehe 1995; Alshuth et al. 2002; Wang et al. 2002; Abraham et al. 2005; Saintier et al. 2006; Ayoub et al. 2011, 2014; Tee et al. 2018)
	Energy-based criterion (*SED*)	- Widely used to assess fatigue failure through energy absorption or dissipation - Direct proportionality between energy release rate and both *SED* and crack size	- Not unify uniaxial and equiaxial tensile behaviour - Unable to predict specific performance such as uniaxial and shear performance - Unable to analyse crack orientation of nucleated cracks	- (Ayoub et al. 2014; Tee et al. 2018)
	Energy-based criterion (*CED*)	- Calculates energy release rate and identifies crack nucleation and failure mechanism under multiaxial loading	- Requires further research for broader use	- (Mars 2002; Harbour et al. 2008; Ayoub et al. 2011, 2012; Zine et al. 2011; Nyaaba et al. 2019a)
Crack growth	Critical plane analysis (*CPA*)	- Accurately predicts fatigue failure and crack growth using *CPA* algorithm and energy release rate analysis	- Detailed modelling approaches are required to assess different fatigue failure conditions	- (Mars 2002, 2021; Mars and Fatemi 2003a, b; Barbash and Mars 2016; Mars et al. 2019)
	Extended finite element (*X-FEM*)	- Models crack growth without requiring mesh adjustments, maintaining mesh consistency	- Limited applications compared to other methods - Requires further development for broader applications	- (Stolarska et al. 2001; Sukumar et al. 2003; Gigliotti and Kroon 2012; Behroozinia et al. 2016, 2019)
	Cohesive zone modelling (*CZM*)	- Simulates crack initiation and propagation - Able to model separation between two adhesive zones	- Susceptible to mesh density issues, - Negatively affecting computational efficiency	- (Li and Hoo Fatt 2015)
	*J-Integral*	- Calculates energy release rate and identifies crack growth and failure mechanisms	- Neglecting new crack surfaces - It can lead to stress singularity problems	- (Li and Hoo Fatt 2015; Bhattacharyya et al. 2023)
	Virtual crack closure technique (*VCCT*)	- Determines energy release rate equivalents and evaluate crack growth	- Failure locations and crack propagation directions can vary, as the technique focuses on crack mechanism in only one direction	- (Kim et al. 2005; Harper and Hallett 2010; Liang et al. 2021)

## Finite element models

### Studies at material level

Tyre materials are generally classified into two categories: pure rubber components and composite-reinforced rubber components. To date, the numerical approaches discussed above have primarily been applied to understand the performance of tyre components in finite element simulations. As tyre design becomes increasingly sophisticated, advanced analysis methods are gaining importance for practical applications. Consequently, several studies have been conducted to estimate potential damage in tyre materials and applications.

#### Rubber materials

Rubber is widely used in various antivibration applications, such as tyres, engine mounts, and bumpers, due to its elastomeric properties, making it a key component in tyre design. Previous numerical fatigue studies have focused heavily on rubber materials to prevent fatigue failure in tyre components during service. Bin et al. (2014) investigated the fatigue performance of filled natural rubbers in uniaxial tension, focusing on various strain-based damage parameters (including the peaks of the first principal Green–Lagrange strain, Almansi–Euler strain, engineering strain, logarithmic strain, stretch ratio, peak octahedral shear strain, and strain energy density peaks corresponding to the loading and unloading paths of the stress–strain curves) and specimen geometry (a dumbbell simple tension specimen (STS) a dumbbell cylindrical specimen (DCS) and a hollow cylindrical specimen (HCS)). They noted that different fatigue life models can yield different fatigue life estimations. Additionally, selecting a suitable fatigue life model in FE analysis is important, not only for accuracy but also for reducing experimental costs. The general applicability of the *SED* method to simulate uniaxial fatigue behaviour was explored, with the Mooney-Rivlin constitutive model applied to uniaxial test specimens in Abaqus. A strong correlation (R^2^ > 0.95) was reported between the observed lifespans and the predicted lifespans using all the damage parameters. The model with peak of the maximum (1st) principal Green–Lagrange strain showed the highest agreement with the actual fatigue life.

More recently, Belkhira et al. (2020) experimentally and numerically investigated the fatigue life of rubbers, focusing on multiaxial behaviour. Based on the experimental findings, FE models were developed using the generalised Rivlin-law (order 3). These models incorporated 5 damage parameters, including the principal strain, principal stretch ratio, *SED*, and Cauchy-Green deformation tensor and Piola-Kirchoff stress tensor as input parameters to characterise the *CED* parameters. The damage parameters were assessed to evaluate their effectiveness in relating tension and torsion fatigue test results (Table 2), and the strong correlation was found with *CED* (R^2^ = 0.7).

**Table 2:** Comparison of correlations for the predicted fatigue life of NR rubber based on the damage parameters (Belkhiria et al. 2020)

Damage parameter	Correlations (R^2^)
Maximum principal strain	0.65
Maximum principal stretch	0.57
Maximum Piola–Kirchhoff stress	0.66
*SED*	0.37
*CED*	0.7

Verron et al. (2006) introduced the initial step in developing a new multiaxial fracture criterion for rubber-like materials. Their proposed model utilised the framework of Eshelbian mechanics, assuming a uniform distribution of intrinsic flaws in elastomers and employing the properties of the energy–momentum tensor to formulate the criterion. The model was implemented using the UVARM subroutine in Abaqus, which allows to access material point quantities and use them to define user output variables (Abaqus 2013). The study investigates four criteria for crack nucleation: maximum principal stretch, strain energy density, cracking energy density, and the proposed method. The global stretching level was defined to range from 1.0 (representing the undeformed configuration) to 3.0.

**Fig. 7:**
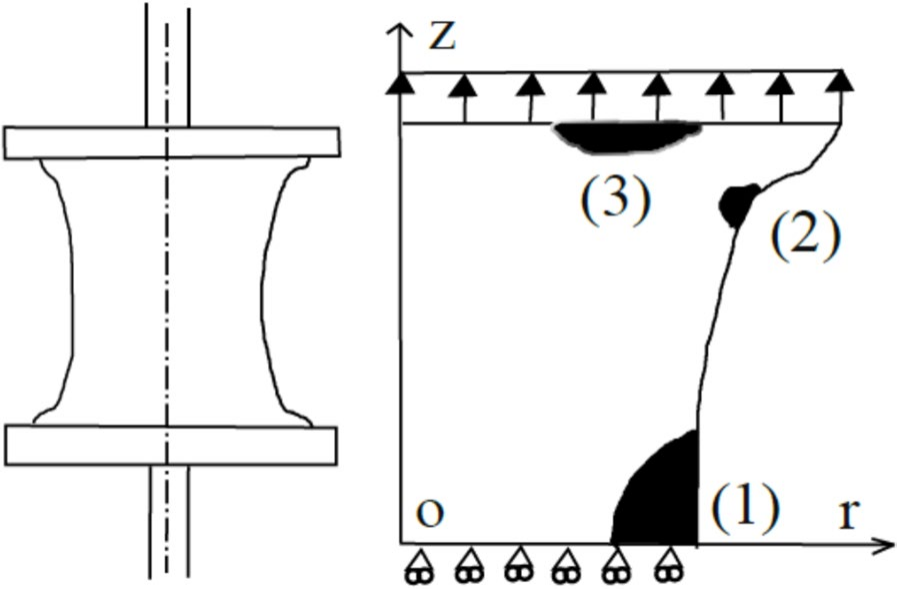
Schematic representation of a dumbbell specimen and location of critical damage zones (1, 2, and 3) observed in the model developed by Verron et al. (2006)

As shown in Fig. 7, three critical zones were examined in a dumbbell-shaped sample: (1) the zone where macroscopic cracking occurs, (2) the curved region near the base of the sample, and (3) the area near the metal base under various levels of stretching. Using the first three criteria (maximum principal stretch, strain energy, and cracking energy density), the damage appears in zone 1 and zone 2 at low stretch levels (around 1.1). Additionally, zone 1 becomes the most damages area, while zone 2 shows no damage at higher stretch levels (around 3.0). The proposed model provided different results compared to the first three criteria. It was found that damage first appears in the curved regions (zone 2) at low stretching levels (1.1), followed by the development of internal cracks near the metal base (zone 3) at moderate stretch levels (1.2–1.4). Finally, macroscopic cracks occurred at the mid-height of the sample (Zone 1) at high stretch levels (above 1.7). This study introduced a preliminary criterion but left key issues yet to be addressed. Further investigation with analytical solutions and industrial applications is necessary, along with a better understanding of the eigenvector’s physical meaning. Additionally, the criterion’s effectiveness in estimating rubber fatigue life needs to be assessed. Luo (2022) conducted a study on crack initiation under different amplitudes and *R* ratios, defined as the ratio of minimum and maximum stress or strain level in a cycle (*σ*_*min*_*/σ*_*max*_ or *ε*_*min*_*/ε*_*max*_). As existing models were unable to accurately assess rubber fatigue damage across varying *R* ratios, a new approach was proposed using an effective tensile strain criterion in the analytical study. The necessary data was obtained from FE models developed using the Mooney-Rivlin material method. The stress–strain relationship was derived using the Cauchy-Green deformation tensor and Kronecker delta methods. Effective tensile strain was then calculated to define damage, as damage does not occur in compression during cyclic loading in antivibration applications. An S–N curve (Wohler curve) was created to evaluate *R* values, and comparisons showed the advantages of using the effective tensile strain damage criterion for different *R* ratios (ranging from *R* < 0 to *R* ≥ 0). The proposed criterion provided a strong accuracy with a correlation coefficient (R^2^ = 0.85) in S–N curve form for 30 fatigue cases under different *R* ratios. The predicted data showed 96.7% accuracy for cylindrical samples. Additional validation across 90 fatigue cases with 15 loading paths confirmed the reliability of the criterion.

Wang et al. (2014) conducted a series of uniaxial tension/compression fatigue experiments on dumbbell-shaped cylindrical rubber specimens to investigate damage parameters across a wide range of *R* ratios. A hyperelastic material model using Mooney-Rivlin constants was implemented in Abaqus, and critical strain values were identified at critical locations for different *R* ratios. The constitutive material model included the transient softening, associated with Mullin’s effect by employing cyclically stabilised quasistatic stress–strain curves from the fifth loading cycle. Initially, two fatigue life models were evaluated (model I and model II), focusing on peak strain (*ε*_*max*_) and strain amplitude (*ε*_*a*_) as key damage parameters, respectively. The fatigue mean life (*N*_*f*_) data corresponding *R* < 0 (negative strain ratio loading conditions) and *R* ≥ 0 (non-negative strain ratio loading conditions) were derived using power-law relationships for model I and model II (Table 3). Additionally, an alternative life prediction approach was proposed based on an equivalent strain amplitude to predict *N*_*f*_ under *R* = 0 and *R* < 0, and the proposed approach successfully predicted fatigue life over a broad range of *R* ratios with (R^2^ = 0.923). It is highlighted that the proposed approach can be applied to design filled natural rubber for engine mounts, with a fatigue life ranging from 80,000 to 2,000,000 cycles and the strain values − 0.48 (minimum) and 0.81 (peak).

**Table 3:** Strain-based power-law relation for different *R* ratios

*R* ratios	Models	Power-law relation	R^2^
*R* < 0	Model I	${\varepsilon }_{max}=20.91 ({{N}_{f})}^{-0.28}$	0.875
	Model II	${\varepsilon }_{a}=6.90 ({{N}_{f})}^{-0.22}$	0.300
*R* ≥ 0	Model I	${\varepsilon }_{max}=8.40 ({{N}_{f})}^{-0.20}$	0.727
	Model II	${\varepsilon }_{a}=9.56 ({{N}_{f})}^{-0.28}$	0.948

Li and Hoo (2015) presented Charpy impact tension and fracture tests to evaluate the dynamic tearing performance of rubber. *CZM* techniques were implemented using an Abaqus explicit user-defined material subroutine (VUMAT) to assess crack propagation in rubber under dynamic loading. *CZM* material models were developed based on the traction–separation law, incorporating damage characterisation, and the material behaviour before damage, the damage initiation criterion, and the response after damage onset are defined. The hyperviscoelastic behaviour of natural rubber (NR) was modelled, using an equilibrium spring approach and considering the Maxwell element. The Maxwell element comprises a viscous damper and a hyperelastic intermediate spring in series where the total Cauchy stress was derived from both the equilibrium and intermediate springs. The resistance of the damper was determined by the strain rate. Additionally, the Yeoh (third-order reduced polynomial) function was discussed to account for hyper-viscoelastic behaviour. The proposed modelling procedures were found to reliably predict the dynamic tearing performance of rubber, with a finer mesh applied in localised regions around the crack tip and expected crack path. The side-by-side image comparison confirmed that FE analysis accurately replicated the failure progression observed in both the tensile strip tearing test and the shear test. For instance, a comparison of tensile strip tearing test in FE and experimental results for a 76.2 cm drop height showed alignment at key time points: *t* = 0 ms (test start), *t* = 18 ms (crack tip blunting), *t* = 27 ms (fracture onset), *t* = 30 ms (crack growth), and *t* = 80.7 ms (final break). Elmukashfi and Kroon (2012) employed finite-viscoelasticity theory and *CZM* techniques to evaluate dynamic crack propagation in rubber. A nonlinear finite element approach with an implicit time integration scheme was employed to model a crack initiated in a stretched rubber sheet under plane stress conditions. The numerical model accounted for various contributions to fracture toughness, including surface energy, viscoelastic dissipation, and inertia effects.

**Fig. 8:**
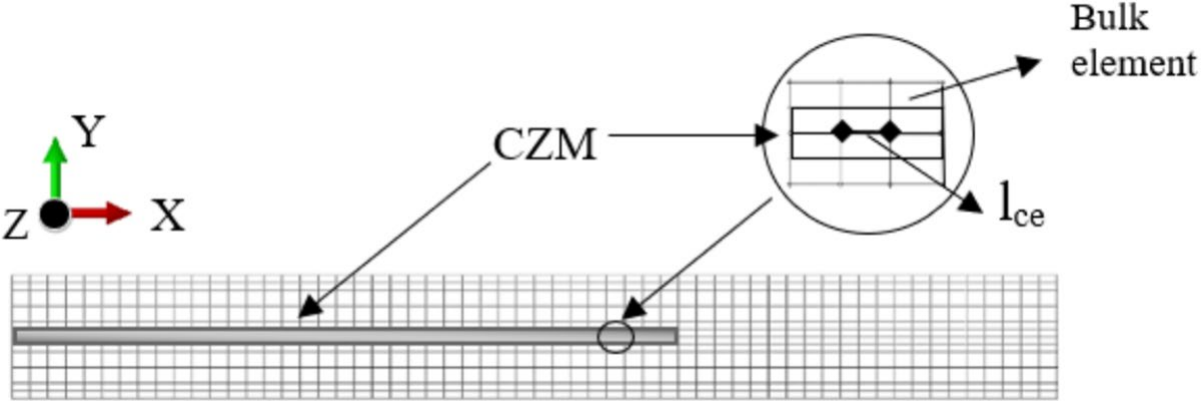
The finite element mesh of the thin rectangular sheet of rubber modelled by Elmukashfi and Kroon (2012)

As shown in Fig. 8, cohesive elements were embedded along the crack propagation path, with the cohesive element length (*l*_*ce*_) set to 0.1 mm, and the cohesive zone parameters applied in Abaqus, including cohesive zone strength (*σ*_*c*_), cohesive energy (*G*_*IC*_), and initial stiffness (*K*_*n*_). It is reported that viscoelastic dissipation in rubber is negligible due to the rapid crack propagation compared to the relaxation time of the rubber. Although the cohesive law was considered rate-independent, viscous effects suggested a degree of rate dependence. Simulating crack growth at low speeds was challenging; lower applied stretches prevented the crack from developing further and reaching a steady propagation state. Consequently, stretches below 3.0 were not examined in this study while experimental data had a maximum stretch of 4.0. It is also highlighted that the fracture energy estimation does not represent the surface energy required for crack formation but also includes significant dissipation near the crack tip. This dissipation occurred in the surrounding bulk material and was not fully captured by the continuum viscoelasticity model. Wu et al. (2021) conducted wear tests on tread blocks, investigating lateral sliding conditions with varying draft angles, root radius, interfaces, and slip angles. In addition, representative numerical FE models were developed. The Yeoh material model was used in Abaqus, and the fatigue performance of the tread blocks was evaluated based on the maximum Green–Lagrange strain (G-L) method derived from FE model results (Eq. (2)).

2 $$\varepsilon_G=\frac{\left(\varepsilon_E+1\right)^2-1}2$$

where *ε*_*G*_ Is the maximum G-L strain and *ε*_*E*_ is the maximum logarithmic strain. The *ε*_*E*_ obtained from FE models were used to represent damage parameters. The FE results were compared to the test results through visual image analysis. The wear failure pattern in each block was found to be similar, and these strain limits captured lateral sliding behaviour observed in the experiments. This approach was recommended for assessing fatigue behaviour. Zarrin and Fattami (2013) conducted research into cumulative fatigue damage and life prediction of elastomeric components. The effectiveness of various damage approaches, including maximum principal strain as a damage criterion, as well as the Rainflow cycle counting procedure and Miner’s linear cumulative damage rule for cycle counting, was evaluated under variable amplitude service loading condition. Note that Rainflow cycle counting is a method used in fatigue analysis to identify and quantify stress or strain cycles under variable amplitude loading. It simplifies complex loading histories by identifying peaks and valleys, counting reversal cycles, and classifying them based on stress range. Meanwhile, Miner’s linear cumulative damage rule assumes that each stress cycle causes some damage, and failure occurs when the total damage reaches 1. The half cradle mount of an automobile was modelled in Abaqus, leveraging the symmetric geometry of the mount and loading conditions. Hyperelastic material properties were applied in the material modelling, while Mullin’s effect was considered to simulate softening behaviour under reversal loading conditions. Maximum principal strain and *R* ratio at the integration point of this element were assessed for each cycle. Initially, the maximum principal strain was obtained for the critical element, followed by calculations for crack initiation and total life predictions. The predicted total lifespan was assessed at two stages: crack nucleation and final failure. The average ratio of predicted based on FE results to experimental results was found to be 3.69 and 0.78 for these stages, respectively. The study reported that total life predictions were more reliable than crack initiation predictions due to the dominant role of macrocrack growth in the overall fatigue life of the component.

Barbash and Mars (2016) evaluated the fatigue growth of rubber during bushing operation by monitoring damage development in a filled natural rubber bushing under road loads. Initially, the material characterisation was performed, followed by the computation of the load-strain relationship through FE analysis. The critical damaging effects of road load history were then assessed using critical plane analysis. This analysis not only identified fatigue-critical areas but also provided crucial insights into tracking crack opening and closing states, local loading history, ranking of damaging cycles, and crack growth orientations, offering a detailed understanding of damage within rubber components. Rainflow cycle counting method was used to evaluate the damage rate in variable amplitude histories. In this approach, if a loading pattern had *M* cycles, the total crack growth was calculated by summing the growth from each cycle. The growth rate was assessed throughout the entire loading pattern to estimate the number of repetitions required for the crack to propagate from its initial size to failure. Finally, normalised total damage contour plots and the physical test results at the same load histories were compared. The predicted fatigue life aligned well with the observed results.

In another study, Wang et al. (2024) explored a thermomechanical coupling approach combined with *CPA* to investigate fatigue nucleation in rubber materials. They reported that the fatigue life of a single element (*Nₛ*) is not representative of the overall lifespan of the bearing and proposed a novel method (*N₀.₅*_*%*_) to predict fatigue life by averaging the lowest 0.5% of elements instead of relying on the shortest-life element (*Nₛ*). Optimised rubber bearing designs were tested and simulated to evaluate their fatigue life performance. The focus was given on the original model, model C and model G (lower and upper-emboss ribs, respectively). The 3rd-order Ogden hyperelastic material model was employed, adjusted using curve-fitting techniques based on experimental test results. Thomas model *(da/dN* = *f (T,R))* was employed to simulate fatigue crack growth, and this model was also used to calculate the *Nₛ*. *da/dN* is fatigue crack growth rate, *T* is the tearing energy, and *R* is the load ratio *R*. To establish the fatigue parameters of Thomas model, fatigue crack growth rates were tested at different energy levels. The power-law self-heating model was adopted to simulate the heat accumulation behaviour of rubber, with material parameters obtained through this model using micro-kinematic hysteresis from the Endurica CL code. It is highlighted that the *N*_*₀.₅%*_ model overestimates absolute fatigue life (Table 4), while its key contribution lies in providing a more stable and reliable trend prediction for different designs. Authors suggested that *N*_*₀.₅%*_ requires an empirical scaling factor for accurate life predictions but could be used as a tool for guiding design optimisation by minimising numerical errors caused by localised stress concentrations.

**Table 4:** Comparison of experimental and predicted fatigue life of rubber bearing studied by Wang et al

Model	Experimental life (cycles)	Numerical life (cycles)	Experimental/numerical (cycles)	*N₀.₅%* (cycles)
Original model	22,231–37,235	18,440	1.21–2.02	229,800
Optimised model C	75,904	85,550	0.89	511,800
Optimised model G	80,597	33,680	2.39	487,800

#### Composite components

**Fig. 9:**
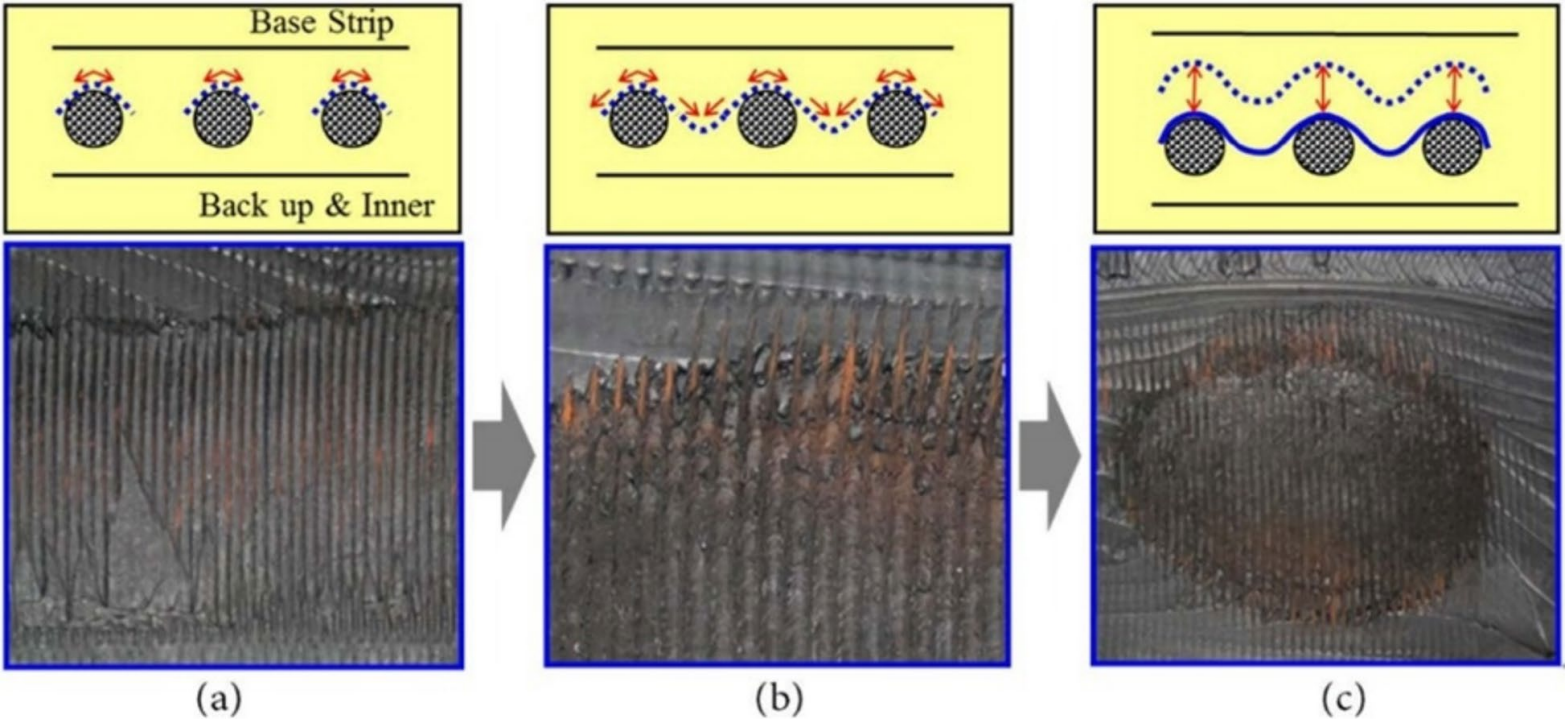
The process of carcass ply separation of radial tyre. **a** Threshold, **b** crack growth, and **c** failure (Jeong et al. 2019)

Reinforcement strategies using nylon-based reinforcements, steel cords, or multi-layered designs provide the strength, stiffness, and overall structural integrity to tyres. Belts and plies work as internal reinforcement layers, enhancing tyre durability and service life. Damage to these belts or plies can result in irregular wear patterns, internal damage, reduced tyre performance, and cracks that typically initiate at the belt edge and propagate along the belt-rubber interface (Cho et al. 2015). Additionally, cord-rubber interfacial debonding is one of the primary failure modes encountered in fatigue due to the significant anisotropy between rubber and cord, material imperfections, and manufacturing defects. As the mechanical properties of cord-rubber composites play a critical role in understanding the merits and limitations of interfacial adhesion, which directly affects tyre fatigue life, numerous researchers have experimentally examined the interfacial morphology and mechanical attributes of cord-rubber interfaces (Lee et al. 1996; Shi et al. 2015; Rosu et al. 2016). Previous studies have focused on three primary types of fatigue damage: separation between the fibres and rubber matrix, cracking within the rubber matrix, and cord rupture (Valantin et al. 2015). For example, Jeong et al. (2019) reported that separation between the plies in a multi-ply radial passenger car tyre is a rare occurrence and often seen in bias and bias-belted tyres. Moreover, slight misalignments in radial ply angles rarely lead to separation, though they are typically indicated by sidewall bulges or softness. In radial truck tyres, the steel carcass plies can exhibit visible bulges on the sidewall, indicating potential separation and a future risk of failure. Separations in truck tyre carcass plies often begin with surface rust on the steel cords and progress to complete separation as cracks develop (Fig. 9).

Pidaparti and May (2001) studied the fatigue crack initiation and propagation at the cord-rubber interface using tearing energy (*TE*) and *J-integral* approaches. Two types of cracks were investigated: crack I, which propagates along the interface between the cord and rubber, and crack II, which is normal to the interface between the rubber and cord materials. A micromechanical model in Abaqus was used to simulate the stress distribution and crack propagation behaviour in cord-rubber materials under cyclic loading. Then, *TE* and *J-integral* methods were used to evaluate the strain energy (*SE*) release rate, quantifying the energy required for crack propagation at the cord-rubber interface and compared in both 2D and 3D models. the *TE* and *J-integral* methods were analysed. Note that both the *TE* and *J-integral* methods rely on energy principles, but they employ different calculation techniques. For crack type 1, the results of the *J-integral* and *TE* methods were analysed under linear and nonlinear loading conditions using 2D and 3D models. In 2D analysis, the results were nearly identical at a 20% strain level. However, the 2D linear analysis predicted *J-integral* values that were 154% higher than *TE*, whereas the 2D nonlinear analysis indicated that the *J-integral* was 161% higher than *TE*. For 3D analysis, the *J-integral* values were found to be 16% higher than *TE*, while in nonlinear analysis, the difference increased to 22%. For crack type 2, *J-integral* comparisons were conducted for both linear and nonlinear analyses in 2D and 3D models. The 2D models predicted significantly higher values than the 3D models, with differences of approximately 261% at 5% strain and 390% at 20% strain. Additionally, when comparing linear and nonlinear analyses, the 2D nonlinear analysis consistently predicted lower *J-integral* values than the linear analysis. At a 20% strain level, the linear analysis predicted values 20% higher than those obtained from the nonlinear analysis. Crack size comparison using the *J-integral* method showed that 2D linear values were higher than 2D nonlinear and 3D linear values. For crack 2, 2D linear was about 280% higher than 3D linear at 20% strain. The authors confirmed that crack size significantly affects *J-integral* values and fracture toughness predictions in cord-rubber composites.

Li and Xin (2018) investigated the flexural fatigue performance of V-belts made from fibre-reinforced rubber using a combination of FE material modelling approaches, including hyperelasticity, viscoelasticity, and the Mullins effect. The research highlights that adding short fibres enhances the belt’s lateral stiffness but reduces its flexural fatigue performance due to stress-softening (Mullins effect) and fibre-matrix slippage. V-belts are the most frequently used power transmission belts in the rubber industry. However, when these belts operate under periodic cyclic loads, their efficiency gradually decreases due to fatigue damage (Fedorko et al. 2013; Honus et al. 2017). The De Mattia test is a widely used method in the rubber industry for evaluating the fatigue behaviour of rubber materials, focusing on the material’s resistance to crack initiation and growth under repeated flexing (ASTM 2000).

A De Mattia test was used to study crack formation and extension. To simulate fatigue behaviour, a combination of standard and explicit solvers was employed in FE models. The standard solver was used for the belt tensioning stage while the explicit solvers was used for the dynamics of the transmission system. Additionally, a simplified representative model was modelled to examine the De Mattia rubber flexural fatigue performance. A Neo-Hooke model, along with an additional mathematical model, was proposed to account for viscoelastic behaviour, while the Mullins effect was incorporated based on cyclic uniaxial tensile properties. *SED* was identified to assess key main factors in fatigue damage, and empirical fatigue life calculation was developed. The predicted fatigue lives are given in Table 5. The study concluded that FE analysis-based approach was found to be reliable, and the comparison between predicted and experimental fatigue life results showed good agreement. Luo et al. (2023) conducted an extensive numerical and experimental study on the fatigue performance of cord/rubber laminates, focusing on interfacial adhesion and the fatigue behaviour of the rubber matrix. Experimental programme included pulley/bending fatigue tests, cord/rubber adhesion tests, fatigue crack growth tests, and uniaxial tensile fatigue tests to analyse fatigue behaviour characteristics. Two life prediction approaches were evaluated: the crack growth approach and the crack nucleation approach. A global modelling strategy was employed to determine the interfacial mechanical properties of cord/rubber laminates under multiaxial loading while capturing the large-scale deformation and load distribution. Also, a local modelling strategy was used, focusing on the critical regions identified through the global model. This approach provided a microscale understanding of the interfacial mechanical properties, including interfacial stress and strain distribution, crack initiation and propagation mechanisms, and adhesion fatigue performance. For example, the pulley/bending fatigue test (S1 sample) was simulated using a multi-step approach, starting with the application of a static load and followed by steady-state transport within the implicit domain in the global analysis, while the local analysis was modelled in 3D with the implicit domain. S1 sample under the cyclic compressive-tensile multiaxial fatigue loading was used to apply crack growth prediction to real-world fatigue conditions. The crack growth rate (*da/dN*) was correlated to the tearing energy (*T*_*max*_) in the local model, with stress and strain distributions extracted from the global analysis. The crack growth behaviour was classified into four regimes: regime 1 (*T*_*max*_ ≤ T_0_), regime 2 (T_0_ < *T*_*max*_ ≤ T_t_), regime 3 (T_t_ < *T*_*max*_ ≤ T_c_, power-law regime), regime 4 (*T*_*max*_ > T_c_, rapid failure). In comparison, *SED*-based predictions were more conservative, underestimating the fatigue life for smaller pulleys. FE results of S2 sample helped to assess different predictors, including *SED*, maximum principal Cauchy stress (*S*_*max*_), maximum principal strain (*E*_*max*_), equivalent tensile strain (*E*_*equiv*_), and the *CED* in crack nucleation analysis. For S1 sample, several predictors were tested, including *SED*, *S*_*max*_, *E*_*max*_, *E*_*equiv*_, and *CED*. For a 12.5-mm pulley diameter, the experimental fatigue life was about 2 × 10^4^ cycles, and *CED*-based predictions were slightly lower but still well aligned with the experimental data. The scatter plot comparing predicted and experimental fatigue life demonstrated in the paper. It was found that the *CED* predictions closely aligned with the experimental fatigue life values and provided the most reliable estimation. For crack growth predictions, S3 sample (pure shear fatigue growth test sample) was modelled to measure crack growth in rubber.). When *T*_*max*_ = 0.15 kJ/m^2^, the experimental fatigue life was ~ 10⁷ cycles, and predictions reached ~ 10⁹ cycles, overestimating by a factor of 10. The results showed that *T*_*max*_ based predictions overestimated fatigue life. It is highlighted that the established S–N curves in the literatures based on some macro predictors with displacement and force cannot be representative to estimate lifetimes of different types of cord/rubber laminates under multiaxial loading conditions, and an appropriate predictor is important to capture accurate life prediction. The number of numerical studies on the fatigue behaviour of tyre components remains limited, and specific modelling approaches are essential for predicting tyre performance under fatigue loading, particularly for sectional design. To validate these approaches, a comprehensive database is required, including configurations that represent a wide range of material parameters, cross-sections, and loading scenarios.

**Table 5:** Experiment fatigue life and predicted fatigue life under different spacing of pulleys (Li and Xin 2018)

Spacing of pulleys (mm)	Experiment fatigue life (N_e_) of V-belt (h)	Predicted fatigue life (N_p_) (h)	N_e_/N_p_
260	4.16	5.17	0.80
262	8.33	8.81	0.95
264	10.55	9.1	1.16
266	17.22	12.4	1.39
268	18.33	13.87	1.32
270	33.88	22.51	1.51
272	42.77	30.93	1.38

Material characterisation plays an important role in determining the fatigue performance of entire tyre structure, and material-level models capture critical input parameters for system-level simulations. Localised characteristics such as critical strain levels, stress amplitude distributions, and crack developments directly affect damage accumulations and crack propagation path in tyre structures. Therefore, a reliable understanding of material modelling techniques is necessary to accurately predict the fatigue life of tyres under real-life service conditions.

### Tyre applications

The numerical material techniques for tyre components under fatigue loading, as discussed earlier, directly influence the structural response of full-scale tyres. Depending on the type and composition of the tyre components, the nonlinear material response can either reduce or enhance the fatigue strength compared to components made purely from elastomer or composite materials. Multiscale progressive failure, which uses multiple failure criteria to design a tyre, has also been studied. Current and emerging design approaches that capture this response are reviewed and evaluated for full tyres herein.

Wang et al. (2021) conducted experimental and numerical studies to investigate fatigue life performance of passenger car tyres (175/75R14 model), focusing on possible crack propagation in three key regions: the carcass, apex (rubber component between the bead and the sidewall), and abrasion around the bead region. Fatigue life parameters, such as *ΔSED*_*max*_*-N*_*f*_ (maximum strain energy density range and the number of cycles until failure), were initially defined through experimental testing at the compound level. A numerical study followed, using Yeoh material modelling techniques and steady-state rolling analysis in Abaqus. After calculating *ΔSED*_*max*_ from the numerical study, the *N*_*f*_ values (applied cycles until failure) were calculated for three compounds: carcass, apex, and abrasion, respectively. Then, *D*_*f*_ (driving distance until failure) were determined using the formula ${D}_{f}=2\pi {N}_{f}$ at the tyre bead. It is reported that the carcass region is the weakest area in tyre bead fatigue failure. The results were then compared to fatigue life predictions (*D*_*f*_) from tyre drum testing, showing an error of 1.32.

**Fig. 10:**
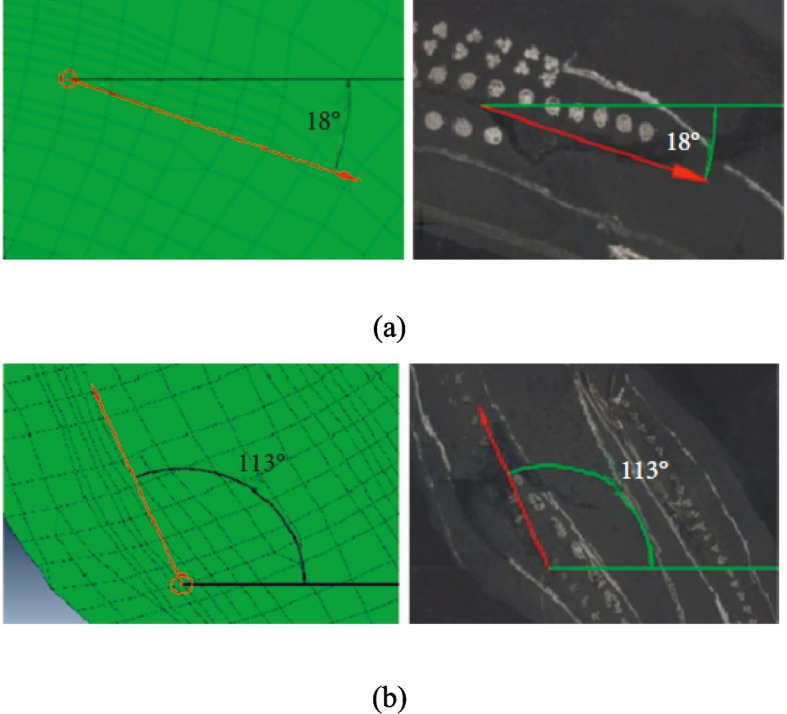
Comparison of failure modes between proposed FE models and test results. **a** Tyre shoulder area. **b** Tyre bead area (Liang et al. 2021)

Liang et al. (2021) proposed a new approach using FE simulation and tyre durability tests to investigate the fatigue failure and life prediction of truck and bus radial tyres based on the *SED* gradient. The challenges of *VCCT* and *CZM* applicability in the model were initially discussed, and the *SED* gradient method was suggested for predicting failure zones and crack propagation in radial tyres. The rubber was modelled with the Yeoh material formulation in Abaqus, while rebar elements were used to represent the orthotropic elastic properties of the cord-rubber system. The study found that the highest *SED* gradient modulus was correlated with the initial failure zone and inversely impacted tyre fatigue life, aligning with the direction of crack propagation. A contour mapping method was employed to identify the fsailure mode position in (*x*, *y*, *z*) coordinates based on the numerical results, demonstrating that the *SED* gradient method effectively predicts damage. FE and test results were compared based on the failure locations and crack directions, and FE results showed strong agreement with test results (Fig. 10). The study also highlighted that the width of the belt was the most critical factor, significantly affecting the *SED* gradient modulus at the shoulder. Additionally, the width of the belt, the interlayer height of the carcass turn-up edge, and the height of the steel wire-reinforced layer were the most influential parameters for the bead *SED* gradient modulus. The findings showed that modifying these structural parameters can improve tyre durability. Therefore, the *SED* gradient method was recommended for evaluating and predicting tyre fatigue failure modes and fatigue life.

**Fig. 11:**
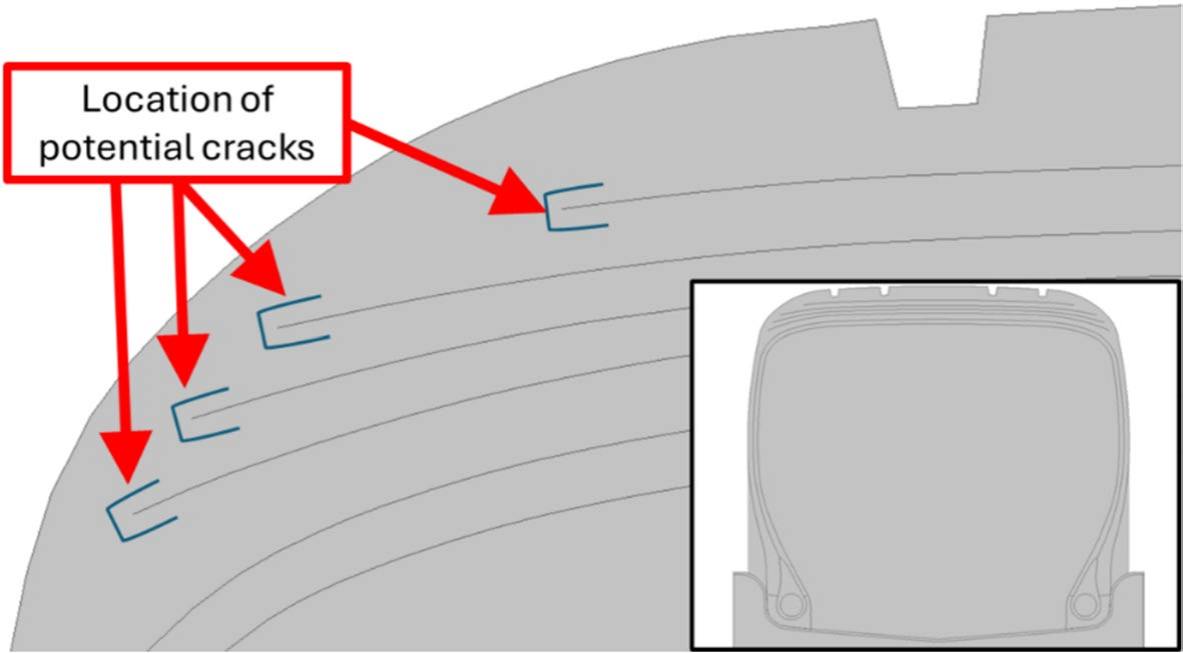
Possible crack tips at the edge of the belt in pneumatic tyres

**Fig. 12:**
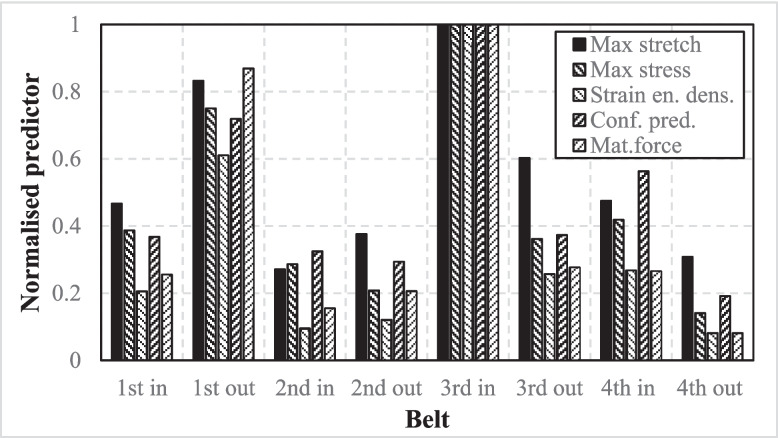
Comparison of the results for the different predictors at belt edges. Authors normalised each predictor with respect to its maximum value (Previati and Kaliske 2012) (note that: inner and outer side of belts were numbered, respectively, in the graph.)

Previati and Kaliske (2012) investigated the fatigue life of pneumatic tyres, focusing particularly on belt separation, where cracks propagate within the rubber between the belts (Fig. 11). A full-scale rolling tyre was examined, using a steady-state rolling FE models and fracture mechanics approaches. All key tyre components including belts, casing ply, rings, tread, and rubber elements were modelled. The Yeoh material model was used to define the hyperelastic properties of the rubber, while reinforcing fibres were represented using 1D rebar elements embedded in the bulk rubber material. Two load cases were considered: vertical load and a combined vertical and lateral load, along with steady state rolling and internal pressure. The most critical failure zones using the simulation match with experimental observations were presented, and crack nucleation as well as crack growth were examined. Predictors were developed using continuum mechanics (maximum stretch, maximum stress, strain energy density, and configurational methods) as well as fracture mechanics (material forces analysis). All predictors were assessed at the same critical locations and normalised values were discussed (Fig. 12).

**Fig. 13:**
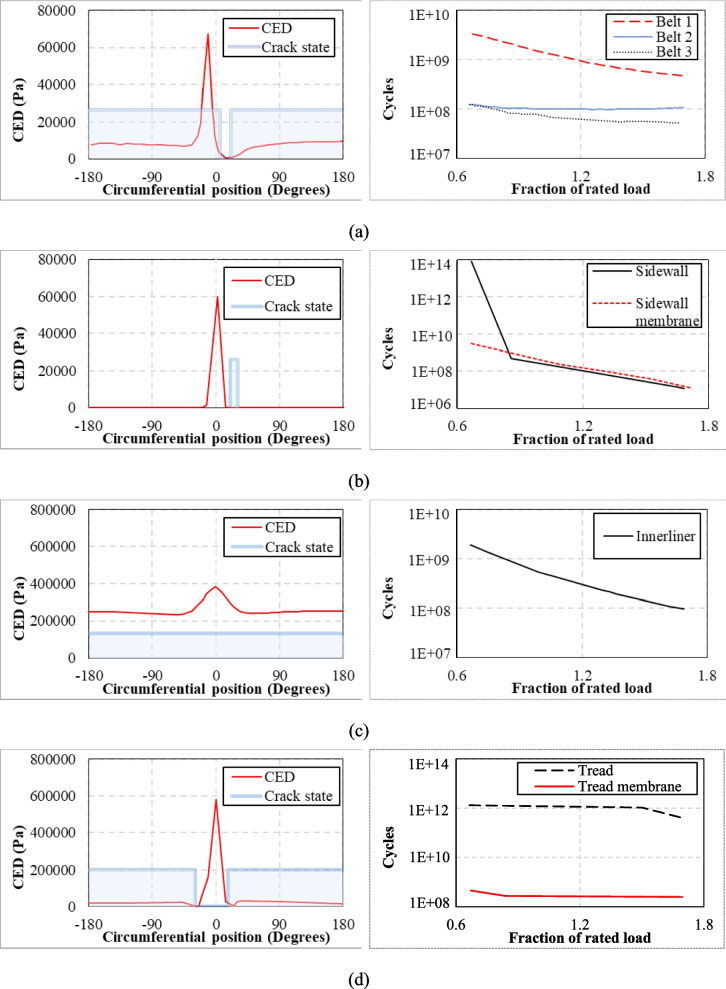
Fatigue durability analysis of tyre components with Endurica CL: **a** belt package; **b** sidewall; **c** inner liner; **d** tread in radial tyres (Mars et al. 2019)

The findings revealed that the most critical zone is the rubber near the inner edge of the third belt. It is also reported that maximum stretch, maximum stress, and strain energy density predictors did not include the damage accumulation overload cycles; however, more detailed failure predictions were captured by using configurational methods and material forces analysis. Nyaaba et al. (2019b) employed a numerical fatigue life prediction framework, integrating FE analysis, experimental material characterisation, and fatigue life prediction models. Thermomechanical fatigue analysis with full 3D tyre was conducted to simulate the tyre’s operational behaviour. The lower sidewall, belt endings, and inner tread lug corners were examined as the most critical regions for fatigue crack initiation. A 2D axisymmetric model of a 56/80R63 ultra-large tyre was model for initial assessment, using Abaqus/Explicit FE software tool. The structural components (rim, bead bundle, belt layers, and casing steel cords) were modelled, while reinforcement layers were simulated employing rebar layers in surface elements. A static tearing energy approach for fatigue crack growth rate, along with Ogden hyperelastic and viscoelastic material modelling techniques, was used. Three main analyses, namely quasistatic loading (deformation analysis), thermal, and fatigue, were conducted for tyre durability prediction, with damage calculations for each material plane and total crack growth being discussed. For deformation analysis, the tyre model under rolling conditions were simulated, and multiaxial strain histories were identified for each component. For thermal analysis, the viscous dissipation energy was extracted for every element. Finally, Endurica CL solver was used to predict fatigue life using the *CED* predictor and rain flow counting algorithm. The study reported the tyre’s fatigue performance under various operating conditions, particularly pressure, vertical load, and speed. Note that, the FE models did not replace direct experimental validation of full-scale tyres, and the study calibrates the FE model using material characterisation tests and previous papers on fatigue damage mechanism to support its findings. Parametric studies were also conducted, including tree inflation pressures, two vertical loads, and two rolling speeds as well as summer (28.9 °C) and winter (0.56 °C) conditions. High damage accumulation, primarily due to weak bonding between cords and rubber and high tensile strain, was observed at the belt endings, while the lower sidewall region was more prone to damage due to heavy rim loads and high thermal stresses. The corner nodes of the tread lugs exhibited high stress intensity and low fatigue strength in these areas. Nyaaba et al. (2019a) investigated the fatigue life of mining dump truck tyres (CAT 795F), focusing on the belt package. The study was conducted under operational conditions at a coal mine in the USA. The *CPA* method was used while assessing the effect of inflation pressure, axle load, and speed on fatigue life. The researchers began with material characterisation, emphasising critical regions of the tyre, such as the apex (bead filler), belt, inner liner, sidewall, and tread. A series of mechanical tests, including stress–strain, stress relaxation, and fatigue crack growth, was performed to obtain the necessary properties. To simulate a 3D treaded FE model, Ogden hyperelastic modelling was employed in Abaqus for each compound, while a Prony series approach was used to model viscoelastic behaviour. Note that a partial experimental validation with FE results was done using material and crack growth tests, and the study did not include a full-tyre rolling drum test for FE comparisons. FE-predicted tyre fatigue life comparison was made according to the given coal mine average tyre replacement life. The critical plane analysis was conducted using Endurica CL’s algorithm, which identifies that the shortest fatigue life via a critical plane search for the revealed that the belt edges were the most critical areas for both crack initiation and propagation. Significant damage accumulation was identified in the belt region, attributed to weak bonding between the cords and rubber. The researchers also assessed the impact of inflation pressures at various levels, discovering that increased inflation loads in strain-crystallising rubber parts influenced crack growth. Higher axle load led to crack nucleation and crack growth at belt edges. Mars et al. (2019) investigated the fatigue durability performance of truck and bus radial tyres under varying loads, including key characteristics such as crack growth rate, crack precursor size, strain crystallisation, and fatigue threshold from a fracture mechanics perspective. FE analysis and Endurica CL fatigue solver were used to develop the fatigue analysis approach. The Neo-Hookean stress–strain law was applied to define the rubber’s hyperelastic material properties, while rebar elements were used to model the cords within the tyre in Abaqus. Initially, FE showed how stress varied across different orientations and demonstrated the critical locations (belt endings, sidewall curvature, tread grooves, and inner liner) for crack initiation. The study calculated the number of cycles required for crack propagation in different orientations, identifying the most critical plane with the shortest fatigue life using Endurica CL fatigue solver. Figure 13 shows the crack states and *CED* values for different locations. Crack states were defined when a crack is open (under tensile stresses) and closed (under compressive stresses). For example, the circumferential position represented a specific location along the tyre’s rolling path. Zero degree was the centre of the footprint, while + 180°/− 180° was the opposite sides of the tyre rotation cycle. Additionally, the relationship between cycles and fraction of rated load (the percentage of the maximum recommended load capacity) was illustrated. The figures offered a detailed insight into predicting the timing and locations of crack growth as the tyre rotates. Two key points were emphasised: (1) the maximum stress experienced by the crack occurs at the centre of the footprint, with the crack remaining open during most of the rotation of the tyre, only closing upon reaching the footprint; and (2) additional load mainly influences the length of the footprint where cracks are closed. Although thermal effects were not considered, the study reported cracks most often initiated at the steel belt ends, which had the shortest lifespan under typical operating conditions. For the sidewall, cracks were observed to form initially on the outer surface, particularly near the area with maximum curvature due to bending. In the inner liner, cracks were predicted to develop near the toe of the tyre, where it contacts the rim. Tread cracks were first observed at the base of the outboard grooves, and it was noted that the tread’s durability was largely unaffected by applied load until the load reached high levels.

Behroozinia et al. (2018) proposed a new multiscale progressive failure approach to predict different failure types (matrix cracking, delamination, and fibre failure) in cord-rubber composites at the belt region. The study evaluated intelligent tyres (advanced tyres with embedded sensors), focusing on tyre-road contact parameters by observing the interaction between the tyre and the road. The effects of fibre volume fraction, void volume fraction, and degraded cord-rubber composite stiffness on tyre behaviour were investigated. The researchers suggested using micro crack density to account for stiffness reduction in transverse plies through matrix crack initiation and propagation. The failure criteria were assessed based on maximum stress or strain using commercial codes like MCQ Composite and GENOA (Chamis et al. 2013). In GENOA, progressive failure was evaluated using distortion energy, while MCQ Composite classified failures into three categories: matrix cracking, delamination, and fibre failure. Other tyre components, such as rubber, carcass, and calibrated cord-rubber composites, were modelled in Abaqus, considering hyperelastic, viscoelastic, and elastic properties. Loading steps were applied in multiple stages, with a steady state analysis performed in the final stage to assess tyre performance under real loading conditions. The researchers used accelerometers attached to the inner liner of the tyre to measure its radial and tangential acceleration components. These acceleration signals were then compared with FE models for validation purpose. Radial acceleration showed the changes in tyre’s radius when the tyre entered and existed the contact patch, while tangential acceleration tracked the deformation and interactions between the tyre and roads.

**Fig. 14:**
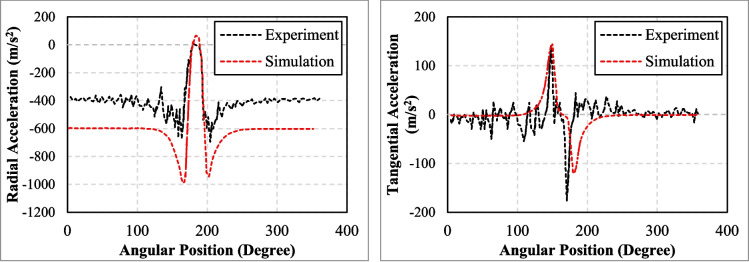
Comparison of simulation and experiment for (**a**) radial and (**b**) tangential components of acceleration signal (Behroozinia et al. 2018)

The comparison confirmed that the FE models had a good agreement with test results (Fig. 14). The researchers also visually compared the acceleration signals from the FE model and the experiment. FE results showed that higher voids lead to more deformation while weaking the composite, and more fibres increased the stiffness and reduced deformation. Conversely, acceleration peaks increased when the fibre volume fraction and fibre direction were reduced.

Moon et al. (2022) conducted a numerical investigation of ultra-high performance (UHP) tyres using steady state rolling analysis under actual driving conditions, rather than focusing on typical tyre lifetime. A tyre lifetime map was produced to estimate fatigue life under different load conditions and driving styles. This model included FE analysis methods, steady-state rolling analysis, *SED* measurements, actual driving patterns, and Miner’s rule for fatigue damage accumulation. UHP passenger car radial tyres of size 215/45R17 were simulated using a 2D cross-section of tyre, and later, the FE model was rotated 360° to form a 3D model. *SED* values were incorporated into the tyre’s fatigue characteristic function, and the driving distance was considered based on the dynamic rolling radius and fatigue cycles. Mooney-Rivlin model (strain energy function) and Ogden model (principal elongation function) were employed to model hyperelastic material behaviour. Note that this study did not conduct and physical experimental tests, and FE results were compared with past experimental studies (Table 6). To predict life prediction, maximum *SED* values were calculated, and the driving distance was considered based on the dynamic rolling radius and fatigue cycles. The analysis accounted for both mechanical fatigue and thermal ageing, with the total sidewall damage assessed using Miner’s rule. The study reported that maximum deformation occurred in the tyre sidewalls, which extended sideways due to the vertical load and internal pressure applied to the tyre.

**Table 6:** Comparison of results from the model developed by Moon et al. with previous studies for validation purposes

Reference	Purpose	Reported value	Predicted value (Moon et al. 2022)
(Oden et al. 1988)	FE validation (rolling contact mechanics)	0.02–0.06 MJ/m^3^	0.0252–0.0339 MJ/m^3^
(Namjoo and Golbakhshi 2014)	FE validation (temperature dependent *SED* values)	Increasing *SED* with higher temperature	Similar increasing trend
(Mars and Fatemi 2003a, b)	Fatigue life data	600M–1.6B cycles	609M–1.58B cycles
(Kim and Jeong 2005)	Rubber fatigue measurements	600M–1.5B cycles	Similar range
(Li et al. 2015)	Tyre-road load conditions (load tests)	4000–10,300 N	5345–9621 N
(Fujigaki and Shimo 2013)	High-speed camera (optical measurement)	Strain: − 0.13 to 0.20	Lower prediction
(Kim et al. 2015)	Actual tyre strain at different load conditions	Strain: 0.1–0.15	Lower prediction

**Fig. 15:**
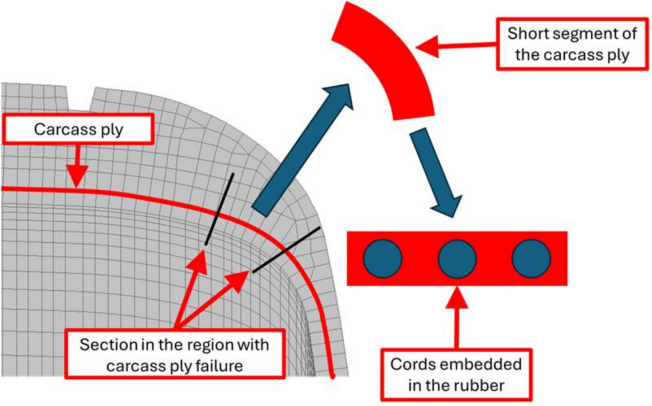
FE modelling strategies employed by Jeong et al. showing areas with different mesh sizes (refined and coarse)

Jeong et al. (2019) assessed the separation in steel cord-rubber composites of radial truck tyres using a combination of global–local finite element modelling. The study highlighted the critical role of the carcass ply in withstanding air pressure and impacts on the sidewalls. 12R22.5 truck tyre components (carcass, ply, belt plies, bead wires, and rubber) were simulated with 3D FE model in Abaqus, and the FE validated using reference papers (Ebbott 1996; Zhong 2006; Jeong et al. 2011). While the simulation results demonstrated good agreement with the experimental failure locations in tyre, a quantitative error analysis was not included in this study. This can be due to the difficulty of precise fatigue life measurements during testing. However, the correspondence of failure locations supported the model’s validity. The penalised first-order Mooney-Rivlin model with a strain-energy function was used to model the rubber, while the cord geometry was modelled based on the spacing and orientation of the rebar cords in the uncured configuration. These geometric characteristics were transferred to the reference configuration of the cured tyre using Abaqus. The global–local technique was employed by refining the mesh in specific sections (local modelling) while interpolating solutions from a coarse global model (Fig. 15). A global model obtained the initial stress distribution under different inflation pressures and vertical loads to assess the cyclic shear strain performance of tyres. Displacement boundary conditions were applied to the local model from the global finite element analysis. The design and performance of various carcass ply profiles (Ver 1–Ver 4) were evaluated in detail, as carcass ply gauge and profile significantly affected overall tyre behaviour. The design versions were described as follows; Ver 1 was the reference design; Ver 2 had an increased topping rubber gauge; Ver 3 was a modified carcass ply profile while changing the curvature and shape of the carcass ply; Ver 4 had an optimised carcass play profile. Failure locations (separation zones) with cut-section image of actual tyre were compared with the FE models. Strain energy, stress, strain, and temperature distribution under inflation pressure and vertical loading were examined. Maximum cyclic shear strain at the interface between steel cords and rubber in the carcass ply shoulder region was thoroughly analysed. The different design versions revealed that changes in carcass profile had a more significant influence on carcass ply separation than changes in topping rubber gauge. It was observed that cyclic shear strain increased with lower inflation pressure or higher vertical loads, with vertical loading having a greater impact on carcass ply separation than inflation pressure. Lastly, steady-state rolling contact behaviour under various driving patterns, including free-rolling, traction, and braking, was analysed, with no notable effect of driving conditions on maximum cyclic shear strain. Note that the paper simulated fatigue loading (the effect of cyclic shear strain); however, it did not perform any fatigue life estimation or simulate progressive crack growth.

Behnke and Kaliske (2018) conducted an advanced numerical investigation into the thermal degradation of steady state rolling tyres under fatigue loading. Heat generation in tyres due to viscoelastic energy dissipation and its effect on temperature-dependent material softening and damage were discussed. A time-dependent thermal damage method (ranging from 0 (undamaged) to 1 (fully damaged)) was introduced, showing how temperature gradually increases over time. The degradation of the material was described in three stages: (i) initial hardening at moderate temperatures, (ii) softening at elevated temperatures due to the mechanical weakening, and (iii) ultimate failure due to polymer chain breakage at extreme temperatures. The study used a 2D finite element (FE) model of the tyre’s cross-section and a 3D FE model based on a 175 SR 14 passenger car tyre. The FE models were calibrated and validated using the experimental results from previous studies (Kratina et al. 2017). Stress at break, strain at break, and fatigue cycles measurements were compared, and FE predicted the similar trends at different temperatures (30°C, 70°C, 110°C) as seen in experiments. For example, the stress at break dropped 0.9% of its original value in experiments while the FE model predicts a thermal damage factor (*d*) = 1.00 at 110 °C. Heath generation, structural weakening, and localised damaged were assessed. The study highlighted cross-sectional damage distribution within specified thermal parameters, identifying potential failure regions, especially in the shoulder areas and reinforcement layers of the belt. The results demonstrated that overheating affects the structural response of the tyre over time, leading to a permanent reduction in overall stiffness. Tyre overheating was also linked to additional damage types, including discrete cracks, delamination between reinforcing cords and the elastomer matrix, cord failure, and abrasion. The researchers concluded that an experimental study is necessary to establish a functional model and quantify the effects of thermal damage on both material and structural scales.

It is important to highlight that the cracking and breaking mechanisms in tyres are rooted in molecular interactions and tend to propagate through critical areas of the tyre. However, monitoring and evaluating these mechanisms during experimental testing can be challenging. As a result, critical regions are often identified after failure, and comparisons between numerical simulations and experimental data focus more on qualitative observations than quantitative data.

## Conclusions

A review of research and practical advances in fatigue failure aspects, tyre components, and tyre applications in the design engineering domain has been presented, and various fatigue failure approaches that are suitable or have potential to design of tyres have also been described. Through an extensive review of previous studies, incorporating advanced numerical analyses, and design considerations, key findings and contributions relevant to fatigue life assessment have been highlighted. Optimising numerical modelling techniques is crucial for improving tyre design, allowing designers to fully harness their potential. The research demonstrates that different fatigue life approaches hold significant potential for structural applications. For crack mechanisms, optimisation is key to the feasibility and efficiency of simulation strategies. Crack energy density (*CED*) is considered the best numerical technique for predicting crack nucleation, especially for multiaxial behaviour, and a growing body of data supports its use for rubber and composite rubber-cord materials. At the crack propagation stage, various methods such as the extended finite element method (*X-FEM*), cohesive zone modelling (*CZM*), the *J-integral*, and the virtual crack closure technique (*VCCT*) have been compared. While these methods provide reasonable predictions of fatigue behaviour, there are still challenges for wider application, as summarised in Table 1. More research and data are needed to refine these techniques and to develop a comprehensive design framework for different tyre components. Practical applications were also explored to improve our understanding of fatigue failure and simulate the performance of full-scale tyres. Studies on various tyre types, including passenger car tyres, truck bus radial tyres, pneumatic tyres, offroad tyres, mining dump trucks, ultra-high performance tyres, and radial truck tyres, have demonstrated the significant impact of the damage under fatigue loading. The findings highlight the potential benefits of current fatigue failure approaches in modelling tyre performance under fatigue stress. Different techniques are applied depending on the predominant failure mechanisms observed in each case. Additionally, the composite cord-rubber compounds, particularly in the belt area, were identified as critical regions in different tyre applications. However, few numerical studies have been conducted, highlighting the need for further research in this area. Overall, this paper reviews current fatigue modelling approaches and their applications to enhance numerical tyre design under fatigue loading. The numerical investigations presented in this paper provide a detailed quantification of tyre fatigue behaviour and for practical design and assessment procedures. These insights can contribute to improving the durability and reliability of tyre structures in real-world applications.

## Data Availability

No new data were created or analysed during this study. Data sharing is not applicable to this article.
